# ID 278 - Canabidiol Adjuvante em Epilepsias: *overview* de revisões sistemáticas e o impacto orçamentário da sua incorporação na perspectiva do SUS do estado da Bahia

**DOI:** 10.5327/2237-9622.2025.v34s1.278

**Published:** 2025-11-25

**Authors:** Manuela Fernandes de Almeida Mello, Bárbara de Castro Santos Silva, Carolina Santos Silva, Gleidson Cardoso Spínola, Rouseli Gonçalves de Menezes, Danielli Nunes de Oliveira Costa

**Keywords:** canabidiol, epilepsia, Avalição de Tecnologias em Saúde, análise de custo

## Abstract

**Introdução:**

As drogas antiepilépticas (DAE) são base do tratamento da epilepsia. A escolha da DAE deve considerar eficácia, efeitos adversos, tolerabilidade e facilidade de uso. A falha no tratamento é considerada após uso de duas DAE (epilepsia farmacorresistente) e atinge 30% dos pacientes. Os análogos da têm sido investigados como anticonvulsivantes na epilepsia farmacorresistente. A Resolução da Diretoria Colegiada (RDC) n.º 327, de 9 de dezembro de 2019, da Agência Nacional de Vigilância Sanitária (Anvisa), concede autorização sanitária para os produtos de para fins medicinais. As síndromes de Dravet (SD), Lennox-Gastaut (SLG) e o complexo da esclerose tuberosa (CET) são apresentações graves de epilepsia, para as quais o canabidiol (CBD) adjuvante (associado a DAE) foi avaliado neste estudo. O estudo visou avaliar a eficácia e a segurança do uso do canabidiol adjuvante para controle das convulsões em crianças, adolescentes e adultos diagnosticados com síndrome de Lennox-Gastaut, síndrome de Dravet e complexo esclerose tuberosa (CET) e seus custos para o Sistema Único de Saúde (SUS) do estado da Bahia.

**Método:**

As buscas foram realizadas nas bases MEDLINE; LILACS e Embase no mês de abril de 2024. A estratégia combinou população-alvo, intervenção (CBD), sem distinção de comparador e filtros para tipo de estudo (revisão sistemática com metanálise) e publicação até dez anos, sem restrição de idioma e do status da publicação. Com o auxílio do Rayyan, foi realizada a elegibilidade em duas etapas por dois revisores independentes e com discordâncias resolvidas por um terceiro revisor. Identificaram-se 745 publicações e, após exclusões, apenas sete atenderam integralmente aos critérios de seleção, todas as etapas foram detalhadas no PRISMA (). Dados foram extraídos em planilha Excel® para sistematização das informações do acrônimo PICOS elaborado. A qualidade metodológica das revisões sistemáticas foi avaliada pela ferramenta (AMSTAR 2). Para o impacto orçamentário, considerou-se a população-alvo de 2 a 18 anos e que 1% da população baiana possui epilepsia com 30% se farmacorresistentes. O preço médio foi de R$ 0,25 por miligrama de CBD.

**Resultados:**

Os resultados encontrados foram: redução absoluta na frequência das crises convulsivas; aumento de pacientes com ausência de crises; redução ≥50% na frequência das convulsões; redução ≥50% das crises quando associado ao clobazam (CLB); eficácia semelhante para 10 mg e 20 mg/kg/dia; redução ≥50% das crises com queda; melhora na variação da escala S/CGIC; aumento do risco de eventos adversos (EAs); diarreia, sonolência, perda de apetite e elevação das transaminases foram EAs mais comuns; aumento de EAs graves; risco maior de EAs graves na associação CBD e CLB; interações complexas entre CBD e DAE; aumento de abandono do tratamento; e com três cenários e custo mensal variando de R$ 1.271.066,37 a R$ 5.084.265,48.

**Conclusão:**

A revisão sistemática demonstrou que CBD adjuvante obteve resultados clinicamente relevantes para pacientes SD, SLG e CET maiores de 2 anos e farmacorresistentes. Os estudos não demonstraram melhorias significantes na qualidade de vida. O CBD adjuvante foi associado a um aumento importante no risco de EAs totais e graves e descontinuação do tratamento. Diante dos benefícios e das incertezas existentes e da gravidade das síndromes epilépticas, conclui-se que, com o monitoramento adequado, o CBD poderia atuar como terapia adjuvante.

